# 

**Published:** 1881-09

**Authors:** 


### CONTENTS.

### *ORIGINAL:*

PAGE.

The International Homœopathic Convention, . . . . . . . 401

The Penn Medical University: A Fatal Error. Ad. Lippe, M. D., Philadelphia, . . . . . . . . . . . 428

Some Key-notes. C. Carleton Smith, M. D., Philadelphia, . . . 431

The I. H. Association. T. F. Pomeroy, M. D., Jersey City, . . . 437

The Binghampton Homœopathic Medical Association. A. J. Clark, M. D., Secretary, . . . . . . . . . . . 439

Potency Physically Considered. C. F. Millspaugh, M. D., Binghamton, N. Y., . . . . . . . . . . . . 440

Acrochordon Chocoe: Characteristics. C. F. Nichols, M. D., Boston, . 444

### *CLINICAL BUREAU:*

*Podophyl.* and *Borax*—In Diarrhœa. E. B. Nash, M. D., Cortland, N. Y, 445

Clinical Cases: *Cannabis*—In effects of post-partum hemorrhage; *Acid. Mur.*—Diarrhœa; *Natr. m.*, *Canth.*, *Rhus tox.* and *Merc.*—Gleet. E. W. Berridge, M. D., London, . . . . . . . . . 446

PERISCOPE, . . . . . . . . . . . . . 452

### *BOOK NOTICES AND REVIEWS:*

Lectures on Diseases of Women. R. Ludlam, M. D., . . . . 456

A Treatise on the Decline of Manhood, Etc. A. E. Small, A. M., M. D., 458

Wanted: Copies of *January* number of THE HOMŒOPATHIC PHYSICIAN, 458

